# Eating disorders symptoms and excessive internet use in adolescents: the role of internalising and externalising problems

**DOI:** 10.1186/s40337-021-00506-5

**Published:** 2021-11-21

**Authors:** Nika Šablatúrová, Jaroslav Gottfried, Lukas Blinka, Anna Ševčíková, Daniela Husarova

**Affiliations:** 1 Faculty of Social Studies, Masaryk University, Joštova 10, 60200 Brno, Czech Republic; 2 Department of Health Psychology and Methodology Research, Faculty of Medicine, P.J. Safarik University in Kosice, Košice, Slovakia

**Keywords:** Eating disorders symptoms, Excessive internet use, Internalising problems, Externalising problems, Adolescents

## Abstract

**Background:**

Both eating disorders and excessive internet use represent significant health issues for contemporary adolescents. Yet, the link between them has seldom been investigated. We aim to study this association through their common underlying psychological factors: internalising problems and externalising problems.

**Methods:**

A representative sample of 7,083 adolescents (M_age_ = 13.48 years; SD_age_ = 1.32; 50.3% girls) from Slovakia was obtained from the Health Behaviour in School-aged Children (HBSC) project in 2018. Study variables included the Excessive Internet Use Scale (EIU) and the Strength and Difficulties Questionnaire (SDQ). Eating disorders symptoms (EDS) were assessed by SCOFF and selected items from the Eating Disorder Screen for Primary Care (ESP). Data were analysed separately for boys and girls with Structural Equation Modelling.

**Results:**

There was a partial correlation between EDS and EIU (*r* = 0.36 for boys and *r* = 0.29 for girls) after controlling for the internalising and externalising of problems. Internalising and externalising problems were positively associated with EDS, while EIU was only associated with externalising problems. The results were comparable for both genders.

**Conclusion:**

The study provides evidence that, during adolescence, EDS and EIU are related and have a tendency to occur together. Also, they are related even when controlled for their shared underlying psychological factors, namely the emotional and attentional/behavioural difficulties.

**Supplementary Information:**

The online version contains supplementary material available at 10.1186/s40337-021-00506-5.

## Plain English summary

The study found that, in adolescents, eating disorders symptoms are associated with excessive internet use even when controlled for their shared psychological factors. Self-control issues were associated with both the eating disorder symptoms and excessive internet use, while emotional issues were only associated with the eating disorder symptoms. The strength of the relationship was similar for boys and girls, even though the prevalence of eating disorders symptoms was double in girls.

**Supplementary Information:**

The online version contains supplementary material available at 10.1186/s40337-021-00506-5.

## Introduction

Adolescence is a life phase that characteristically includes an increased level of risky behaviour and patterns of behaviour that can have a negative impact on health [1]. During this life period, problematic behaviours, risks, and mental health problems often co-occur due to the shared risk factors, such as internalising tendencies (i.e., emotional) and externalising tendencies (i.e., behavioural) when facing problems [2]. In this paper, we focus on two health risks which are often discussed with respect to current adolescents´ health and well-being—eating disorders and excessive internet use. We study these risks in the context of internalising and externalising problems. Although they both have attracted extensive attention, their mutual association has been thoroughly investigated only in a limited number of studies [3, 4].

Eating disorders are characteristic with eating disturbances and rapid weight shifts and represent a significant health issue with potentially severe consequences [5]. Currently, the prevalence of eating disorders is on the rise [6], and it affects more younger ages than in previous decades [5]. According to prior research, gender is an important factor in eating disorders—girls are more likely to experience body-image dissatisfaction and have more desire to control their weight [7]. Even though the prevalence of specific eating disorders (i.e., anorexia nervosa, bulimia nervosa, binge eating disorder), is relatively low—ranging from 0.3% to 8.7%, varying based on the type, definition, and used methodology [8]. The rates for eating disorder symptomatology and subthreshold forms are even higher [9]. Thus, since our study uses a general representative sample of adolescents, we focus on the symptoms of bulimia and anorexia nervosa (henceforth referred to as eating disorders symptoms, EDS).

Excessive internet use (EIU, also often referred to as internet addiction, problematic internet use) is an umbrella term that represents a range of repetitive impairing behaviours related to the online environment [10], with the characteristic presence of symptoms that are typical for addictive behaviours (e.g., persistent preoccupation, mood changes, difficulties with limiting time online, subsequent conflicts [11]). EIU has been shown to be linked to problematic usage patterns, such as computer gaming [12] and the use of social networking sites [13]. Generally, the usage patterns are different for boys and girls—while boys prefer gaming, girls lean towards using social networking [14]. The prevalence of EIU varies considerably depending on the sample and the measuring tool [15]; however, high-quality studies usually report prevalence at a few percentage points (e.g., 2.6% [13]). EIU was shown to be associated with a rather large spectrum of difficulties, including diminished well-being, emotional and behavioural difficulties, school problems, sleep disturbances, and dietary problems [11, 16].

Several studies have indicated that EIU may negatively affect eating habits [17], and reduce physical activity, and thus increase the risk of being overweight and obese [18]. However, weight-loss and/or internalization of thin-ideal associated with bulimia and anorexia nervosa (the symptoms focused upon in this paper) in relationship to EIU may be less apparent. A meta-analysis [19] found an association between social-networking-site use and the internalization of a thin ideal, suggesting that constant exposure to unrealistic body ideals may trigger body dissatisfaction and the need to modify eating habits [20]. However, according to some studies [21], EDS are not isolated phenomena, and the source of the problem lies in the psycho-emotional and social characteristics of the adolescent. Specifically, EDS and EIU may largely overlap due to the fact that they share risk factors and underlying psychological principles, such as behavioural responses like externalising problems (i.e., impulsivity, lower self-control, ADHD) and emotional responses like internalising problems (i.e., depressive symptoms, anxiety, lower self-esteem). In the case of EIU, lower self-control is associated with being prone to immediate gratification, which may become problematic when met with emotional problems because they increase the need to get relief. Easy gratification (which is a dysfunctional coping strategy) may become habitual and further contribute to the escalation of problems [22]. In the case of EDS, the internalising link has been confirmed in many studies. Specifically, EDS was found to be associated with, or even predicted by, social withdrawal, depressive symptoms, nervousness, loneliness, feelings of inferiority, and guilt [23]. The association of EDS and externalising problems was not investigated to the extent that it has a link with emotional problems; however, it was found to be a factor in bulimic symptomatology and binge- eating disorder [24].

Nonetheless, to our knowledge, no study has jointly examined behavioural (i.e., externalizing) and emotional (i.e., internalizing) problems in relation to EDS and EIU, while also taking into consideration possible gender differences. In this paper, we test such a model with a large-scale representative national sample of Slovak adolescents. We aim to investigate the linkages for the individual internalisation and externalisation of problems in association with EIU and the symptoms of eating disorders, separately for girls and boys, to get a more widespread understanding of how adolescents’ digital life is related to the symptoms of eating disorder and problematic eating behaviour.

## Methods

### Participants

Data were obtained from the World Health Organization (WHO) and its collaborative Health Behaviour in School-aged Children (HBSC) study, which was conducted in Slovakia in 2018. To obtain a representative sample of school children aged 11–16, 140 schools (randomly selected from the list of all eligible schools in Slovakia) were invited. A total of 109 agreed to participate in our survey. The school response rate (RR) was 77.85%. Consequently, a representative sample of 8,405 adolescents in Slovakia was obtained (RR = 56.7%). The sample for this study comprises of 7,083 adolescents (mean age: 13.48 years; 50.3% girls).

Participation was fully voluntary with no explicit incentives. When schools agreed to participate in our survey, parents of all students in the 5th, 7th and 9th grades were informed about the study by the school administration, they could opt-out if they disapproved of their child’s participation. Plus, the pupils themselves were given the opportunity to not participate in the data collection even if their parents consented. The data were collected anonymously with an electronic questionnaire that was administered in the school by a team of trained administrators and in the absence of teachers.

Based on a relatively high rate of missing data for items regarding excessive internet use (approximately 15–18%, we argue the high missing rate was caused by positioning the items near the end of the questionnaire), we decided to exclude all data from respondents who answered less than two out of five respective items. The majority of the excluded cases were respondents who quit the questionnaire before completion. The rationale for this listwise data exclusion was the potentially significant bias of the pairwise SEM estimating method, which requires data to be missing completely at random. This assumption was clearly not met. Because the imputation of data with high missing rates is also prone to bias, we decided to sacrifice the final sample size in favour of a more robust and precise estimation. After this exclusion, the total sample size dropped considerably to 7,083, from 8,405 (almost 16% of the sample). As the rate of missing data for all items was now < 4%, we imputed any remaining missing data via the multiple imputation method, using the proportional odds model and the logistic regression model due to the presumably ordinal or binary nature of certain item scale answers. The imputed dataset used for this study is included in Additional file 1.

### Measures

Data for the present analyses were collected using questionnaires from the standardized research protocols for the 2017/2018 WHO-collaborative HBSC study [25], except for the Excessive Internet Use Scale, which was used only in Slovakia. For this study, we used the following measures (items were reversed so that higher scores represent higher EDS, EIU, externalising problems, and internalising problems, respectively)*.*

*Eating Disorder Symptoms* was measured using seven items. The measure was created using the SCOFF questionnaire, a five-item screening tool designed to identify potential eating disorder pathology and to suggest whether an eating disorder might be present [26] and two items from Eating Disorder Screen for Primary Care (ESP) [27] that are typically used for the screening of bulimia nervosa. Participants were asked if they worry that they have lost control over how much they eat, whether they ever make themselves sick because they feel uncomfortably full, if they believe that they are fat when others say they are too thin, and whether they would say that food dominates their life. The fifth item, “weight loss”, was reformulated because the original weight unit, “stone”, is not used in the Slovak language. Therefore, participants were asked if they lost more than six kilograms in three months. The items used from ESP included questions: ‘Are you satisfied with your eating patterns?’ and ‘Do you ever eat in secret?’ Seven dichotomous answers (i.e., yes/no, scored 1/0, possible sum score range 0–7) were used to estimate a factor score of EDS for each participant.

*Excessive internet use* was measured using the five-item Excessive Internet Use Scale (created within the EU Kids Online network; eukidsonline.net), which was validated in 25 European countries [11]. The scale was constructed to capture the symptoms of the components of the model of behavioural addiction (i.e., salience, withdrawal symptoms, relapse, tolerance, conflict [28]). Using a 4-point scale ranging from *never* (1) to *very often* (4), participants rated how often they had experienced such internet use (i.e., not limited to a specific online application) in the preceding 12 months. Possible sum score range was 5–20 and the scale alpha was 0.79. The answers were treated as ordinal when calculating a factor score for each respondent.

*Externalising* and *Internalising problems* were assessed using a two-factor solution from the Strengths and Difficulties Questionnaire (SDQ; [29]). The two-factor solution (instead of four factors) was used for better application to cases with lower scores (i.e., in a general rather than clinical population) [30]. The internalising subscale contained 10 items to measure emotional symptoms and peer difficulties. The externalising subscale contained 10 items to measure behavioural problems and impulsivity/hyperactivity. Items were rated on a 3-point scale (i.e., *not true*, *somewhat true*, and *certainly true*; possible sum score range 10–30 for each subscale). Cronbach’s alpha equalled 0.68 for the *internalising problems* subscale and 0.67 for the *externalising problems* subscale, which indicates acceptable, albeit poorer internal consistency—similar scores were reported in other studies [30].

### Statistical analysis

Structural Equation Modelling (SEM) was used to assess the relationships among the studied variables in R software, version 4.0.2 (R Core Team, 2020), with package “lavaan” [31]. We predicted EDS and EIU with both externalising and internalising problems and estimated the partial correlation between EDS and EIU. Due to reasonable assumptions, we also tested for measurement invariance for boys and girls, aiming for metric invariance that would allow us to compare standardized regression and correlation coefficients between the genders. Due to the low range of response scales (0–1 for EDS, 1–3 for internalising and externalising problems, and 1–4 for EIU), we treated data as ordinal, used theta parameterization, and weighted the least square mean and variance adjusted (WLSMV) estimator.

First, we fitted four simple measurement models separately, each with only one latent variable (i.e., EDS, EIU, Externalising problems, Internalising problems) and the respective questionnaire items. Based on modification indices, we examined the items. Following this step, we allowed for correlated residual variance between several pairs of items within each scale, based on their phrasing or content similarity. After this procedure, all measurement models (except for the EIU measurement model, for which no correlated residual variance would considerably improve its fit) were significantly improved regarding their fit to the data. We implemented the aforementioned residual covariance terms into the SEM invariance models. We employed model fit evaluation rules based on a recommendation from prior study [32] by evaluating TLI and SRMR indices together and, based on their findings, we consider TLI ≥ 0.95 and SRMR ≤ 0.06 to be a good fit, TLI ≥ 0.90 and SRMR ≤ 0.08 to be an acceptable fit, and TLI < 0.90 and SRMR > 0.08 to be a poor fit. Regarding SEM invariance testing, as suggested in guidelines [33], we set ΔCFI =  − 0.01 to be the maximum acceptable fit impairment. We considered the Likelihood-Ratio test to be unreliable in this case, because of its susceptibility to be biased in large samples. The R script of our analysis is included in Additional file 2.

## Results

Table 1 shows the mean, minimum, and maximum score values, and the standard deviations of key variables for each gender in the original data set. Based on the sum score computed from the five original SCOFF questionnaire items and its established cut-off score of 2/3 [34], 267 (7.8%) boys and 489 (14.2%) girls could be classified as being at risk for an eating disorder—this serves as an approximate assurance that the SCOFF items themselves are performing in an expected way, because the rates in our survey are similar to the prevalence reported in previous studies [6].

**Table 1:** Descriptive statistics of the study variables in the original data set

	*M*	*SD*	Minimum	Maximum
Boys				
Excessive internet use	7.72	3.16	5.00	20.00
Eating disorders symptoms	1.65	1.29	0.00	7.00
Internalising problems	14.48	3.13	10.00	29.00
Externalising problems	16.41	3.23	10.00	29.00
Girls				
Excessive internet use	7.65	2.74	5.00	20.00
Eating disorders symptoms	2.15	1.46	0.00	7.00
Internalising problems	15.77	3.43	10.00	28.00
Externalising problems	16.60	3.35	10.00	28.00

N boys = 2, 891; N girls = 2, 898

### Structural equation modelling results

Regarding measurement models, the EIU and SDQ externalizing problem measures showed a good fit for the data, the SDQ internalizing problem measure showed an acceptable fit, and the EDS measure model fitted the data poorly, see Table 2 for detailed fit indices. We ascribe the poor EDS measurement performance to its screening nature, which resulted in weak factor loading on the items. Surprisingly, the two ESP items showed much better factor loadings than certain SCOFF items. We include the EDS item factor loadings in Additional file 3. Alongside TLI and SRMR for indicating a quality of model fit, we report CFI for assessing a change in fit across invariance models, and also RMSEA as a customary addition.

**Table 2:** Fit indices of study measures and tested models

	*χ*2	df	CFI	TLI	RMSEA	SRMR
Measurement models						
Excessive internet use	92.37	5	0.996	0.992	0.050	0.029
Eating disorder symptoms	246.94	13	0.907	0.849	0.050	0.040
Internalising problems	795.50	33	0.948	0.929	0.057	0.063
Externalising problems	421.76	31	0.972	0.959	0.042	0.044
Measurement invariance testing						
Configural invariance by gender	5282.39	898	0.953	0.948	0.037	0.057
Thresholds invariance by gender	5284.53	903	0.953	0.949	0.037	0.057
Metric invariance by gender	5578.29	931	0.951	0.947	0.038	0.059
Scalar invariance by gender	6559.00	952	0.940	0.938	0.041	0.060

We tested the model for invariance across both genders. First, we tested for configural invariance—whether the pattern of factor loadings is roughly the same. This model showed acceptable-to-great fit for the data, χ^2^(898) = 5282.4, CFI = 0.953, TLI = 0.948, RMSEA = 0.037, SRMR = 0.057. We conclude that the configural invariance has been achieved. Then, we tested for the threshold invariance—whether the binary and ordinal items had identical thresholds. Since the CFI of this model remained practically the same as for the configural model, we conclude that the threshold invariance had been achieved. In the same way, because ΔCFI =  − 0.002, we conclude that we achieved metric invariance—whether or not the factor loadings on items are identical. However, scalar invariance—the equality of item intercepts—was not achieved, ΔCFI =  − 0.11. Table 2 contains further details about the characteristics of the invariance models. Nevertheless, since the model shows metric invariance, we can compare standardized regression and correlation coefficients between latent variables for boys and girls.

**Fig. 1:**
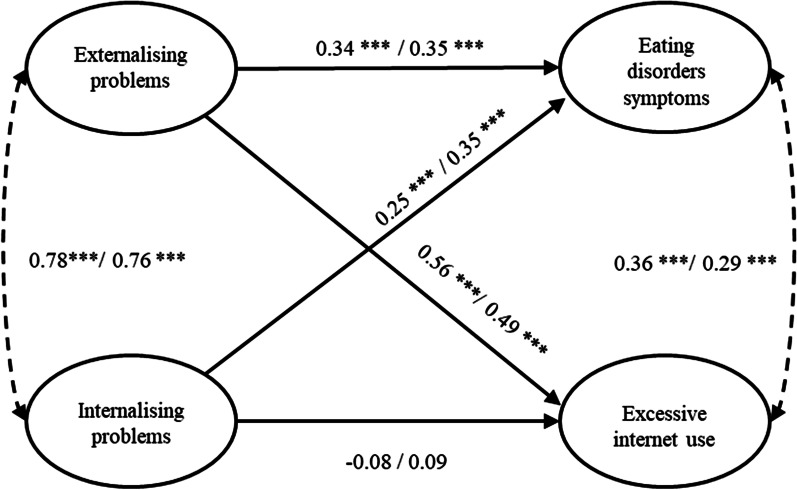
Final model with the standardized regression and correlation coefficients for boys and girls, respectively

When inspected, the mutual latent variable relationships can be considered of more or less equal strength across both genders. In this regard, Fig. 1 shows the standardized regression and correlation coefficients for boys and girls, respectively.

Both dimensions of SDQ were strongly positively cross-correlated (boys: *r* = 0.78; girls:* r* = 0.76) and positively associated with EDS (*β* = 0.34; *β* = 0.35 for externalising problems, *β* = 0.25; *β* = 0.35 for internalising problems, for boys and girls, respectively), but only externalising problems positively predicted EIU (*β* = 0.56 for boys and *β* = 0.49 for girls), while internalising problems did not (*β* =  − 0.08 for boys; *β* = 0.09 for girls). Even after considering these predictors, a weak positive partial correlation remained between EDS and EIU for both boys and girls (*r* = 0.36 and *r* = 0.29, respectively). This partial correlation shows the strength of association after controlling for associations with both the externalising and internalising problems.

## Discussion

In line with the research purpose, this study tested the association between EDS and EIU while taking into consideration internalising and externalising problems as shared factors. Three main findings emerged. First, there remained a partial correlation between EDS and EIU after controlling for externalising and internalising dimensions of SDQ. Second, only externalising problems were associated with both EDS and EIU, while EDS was associated with both externalising and internalising problems. Third, the findings were comparable for both genders, boys and girls.

The first result expands upon the existing literature for the association between EDS and EIU [4] in that these two risks remain partially correlated, pointing to other unexplained mechanisms (not included in the present study) that are responsible for their co-occurrence. We suggest that family-related factors (e.g., parental care, parental monitoring, family dysfunction, family eating habits) might be explanatory factors; indeed, former studies already reported its effects on EDS [35] as well as on EIU [36]. Nevertheless, more research is needed to disentangle the associations between EIU and other forms of EDS.

The second finding points to externalising problems as the only path that relates to both EDS and EIU. Externalising problems is characterised by increased levels of behavioural difficulties, impulsivity, and hyperactivity, suggesting the linkage between EDS and EIU may be enhanced by poorer self-control mechanisms and a tendency to rash action, or sensation seeking [22, 37]. EDS was associated with both externalising and internalising problems, which is consistent with prior literature [37, 38] However, internalising problems lost its effect in the case of EIU, which is not consistent with what had been found before [39]. Nonetheless, it is important to bear in mind that externalising and internalising problems were strongly correlated in this study such that we can presume that, due to the relatively strong effect of externalising problems towards the EIU, the internalising problems lost impact in our model. But that does not mean that emotional difficulties played no role in EIU—rather that they share their effect through the impulsivity/externalising pathway. This indicates that the ‘impulsivity’ pathway dominates in the case of EIU while there may be at least two pathways for EDS (i.e., the impulsive and emotional, since they both kept their effect).

The third result is a novel finding that suggests that the tested associations between the measured constructs were comparable for boys and girls. This is surprising because boys and girls have different patterns of internet use. The most established digital media activity associated with EDS is social media use [4], which has a higher prevalence among girls [14] compared to boys, who are likely to play games [40]. But based on our results, we may hypothetically assume that the associations among the studied constructs are similar regardless of the preferred digital activity. However, the specific online behaviour and the relation of EIU to a specific online application was not measured here and, as suggested [41], the mechanisms, that affect EDS, might vary depending on the specific online activity in which the user engages.

Importantly, there are other limitations that should be noted. The cross-sectional design of this study limits the conclusive statements about the causality between our variables, which could not be established based on our study design. Also, only self-reported questionnaires were used, which could lead to a reporting bias. Furthermore, since data come from an epidemiological survey for which the use of short screening measures is typical, the scale to measure EIU contained only five items and the scale to measure EDS contained only seven items. EDS was measured with the items that consisted largely of that SCOFF instrument that has, akin to other community samples, lower internal consistency, and this has been showed to have rather lower sensitivity in large community samples [42], which was also our case. The EDS measurement used in our study assessed the risk of developing eating problems rather than specific mental health disorders. Moreover, the EIU scale measured generalised excessive internet use and did not assess specific online behaviours; the mechanism of their effect might be modified by the particular facet of the internet [41]. The existence of generalized internet addiction has been criticized and, according to some authors, it is comprised of a combination of various specific internet addictions (e.g., gaming, social networking), rather than an independent concept [43]. However, we did not consider EIU as a separate phenomenon, but as an umbrella term for various internet-based addictions that could not be measured separately in a large-scale epidemiological research project.

## Conclusion

Using a large national representative sample of adolescents with a high response rate, this study confirmed a partial correlation between the EDS and EIU for both genders. This study also showed that externalising problems (i.e., having a poorer control mechanism) are a shared factor for both the EDS and EIU. Therefore, externalising problems in adolescents should be addressed in prevention and intervention efforts.

## Supplementary Information

**Additional file 1:** Imputed data.

**Additional file 2:** R script.

**Additional file 3:** Factor loadings of EDS.

## Data Availability

Data are available with the manuscript.

## Abbreviations

HBSC: Health behavior in school-aged children
EDS: Eating disorders symptoms
EIU: Excessive internet use
SDQ: Strength and difficulties questionnaire
