# Supplementary material for: Eating disorders symptoms and excessive internet use in adolescents: the role of internalising and externalising problems
Source: J Eat Disord. 2021 Nov 21;9:152. doi: 10.1186/s40337-021-00506-5 (PMC8607794; doi:10.1186/s40337-021-00506-5)
Supplement: Supplementary file 1 — Additional file 1: Imputed data. [file 40337_2021_506_MOESM1_ESM.docx]

Appendix: Factor loadings of EDS

| Item | Factor loadings | | |
| --- | --- | --- | --- |
|  | Unstandardized | SE | Standardized |
| Are you satisfied with your eating patterns? (ESP) | 1 | - | 0.43 |
| Do you ever eat in secret? (ESP) | 0.67 | 0.04 | 0.29 |
| Do you believe yourself to be fat when others say you are too thin? (SCOFF) | 0.97 | 0.05 | 0.45 |
| Do you worry you have lost control over how much you eat? (SCOFF) | 1.48 | 0.08 | 0.71 |
| Would you say food dominates your life? (SCOFF) | 0.06 | 0.04 | 0.02 |
| Have you recently lost more than one stone (six kilograms) in a three-month period? (SCOFF) | 0.13 | 0.03 | 0.08 |
| Do you make yourself sick because you feel uncomfortably full? (SCOFF) | 0.26 | 0.02 | 0.25 |
